# The Risk of Hypertension and Other Chronic Diseases: Comparing Smokeless Tobacco with Smoking

**DOI:** 10.3389/fpubh.2017.00255

**Published:** 2017-09-22

**Authors:** Ankit Anand, Md Illias Kanchan Sk

**Affiliations:** ^1^Population Research Centre, Institute for Social and Economic Change, Bengaluru, India; ^2^Department of Population Policies and Programmes, International Institute for Population Sciences, Mumbai, India

**Keywords:** smokeless tobacco, smoking, hypertension, chronic diseases, India

## Abstract

**Background:**

In the past, studies have compared smokeless tobacco and non-tobacco users for the risk of various chronic diseases. The differences in the risk of chronic diseases between smokeless tobacco user and smokers have not been explored. The objective of this study is to estimate the risk of chronic diseases among smokeless tobacco users compared to smokers.

**Methods:**

The data were used from the Study on Global Ageing and Adult Health (SAGE) Wave-1, conducted in 2007–2008 in India. The study sample is the respondents who reported consuming any form of tobacco in last 1 month. The total sample size was 4,038 respondents. The odds ratio of chronic morbidities was estimated taking smokers as the reference category.

**Results:**

The odds ratios for (self-reported) diabetes, asthma, and hypertension were not significant for smokeless tobacco user compared to smoked tobacco users. The odds ratio of chronic lung diseases (CLDs) was significantly lower among smokeless tobacco users compared to smoked tobacco users. The odds ratio of hypertension (measured) combined with low education and belonging to lowest wealth quintiles were not significant for smokeless tobacco users compared to smoked tobacco users. Duration of the use of smokeless tobacco and quantity of use was found to have no significant relation with risk of chronic diseases as compared to smoking.

**Conclusion:**

This study did not find the significantly higher risk of chronic morbidities except for CLD for smokeless tobacco users compared to smoked tobacco users. The study suggests that the use of any form of tobacco may have a similar risk of chronic diseases.

## Introduction

The cardiovascular disease (CVD) is a leading cause of death in India (1, 2). Hypertension is the major risk factor for CVDs and diabetes mellitus (3). Smoking is known for influencing hypertension and chronic diseases, resulting in premature mortality (4, 5). There are a lot of chemical in both types of tobacco. Nicotine is common in both, known to increased blood pressure levels (6). The maximal level of nicotine is similar after a single exposure to smokeless tobacco and smoking (7). Although, smokeless tobacco can cause prolonged nicotine exposure compared to smoked tobacco users.

As per the estimates from Global Adult Tobacco Survey in India, there were 274.9 million tobacco users and out of that 206 million were smokeless tobacco users (8). Smokeless tobacco was responsible for approximately 368,127 deaths in 2010 in India (9). Smokeless tobacco is locally available and sold at a lower price compared to the cigaret and other smoking products and remains the most common form of tobacco use in India (8, 10). As depicted in many studies, the prevalence of smokeless tobacco was high among lower socioeconomic groups; such as in rural and tribal population (11–14). The differences between the smoked and smokeless tobacco use will hold importance for preventing chronic diseases especially among socially disadvantaged groups in India (13). The evidence can be useful in policies preventing the use of tobacco products. Higher taxation, prevention of advertisement of tobacco products and smoking in public places, etc. are more focused on smoking and can continue if smokeless tobacco has a lower risk of chronic diseases compared to smoking (15).

Many studies in the Sweden and United States of America have explored the causal association of CVD risk factors such as hypertension, stroke, and mortality with use of smokeless tobacco (16–19). Haglund et al., Wennberg et al., and Hergens et al. did not find any significant risk of hypertension and stroke (20–22). Hergens et al in Sweden used snuffed tobacco as a smokeless tobacco to study the impact on health (23). Studies have used self-reported hypertension status and duration or amount of smokeless tobacco were not collected. Spit tobacco was found to be associated with increased odds of mortality due to CVDs (24). All these studies that find increased risk of chronic diseases using non-smokers as a reference category. Studies in India have shown that use of smokeless tobacco has increased risks of hypertension and mortality due to chronic diseases compared to non-tobacco users (25, 26). The important question is “does the use of the smokeless tobacco has similar risk of chronic morbidities compared to smoked tobacco?” The objective of the study is to compare the risk of chronic diseases between smoked and smokeless tobacco.

## Materials and Methods

### Data Source

The data were used from the Study on Global Ageing and Adult Health (SAGE) Wave1, conducted in 2007–2008 in India. A multistage, stratified, random cluster sampling design was used. The data were collected from six states—Assam, Karnataka, Maharashtra, Rajasthan, Uttar Pradesh, and West Bengal. The same primary sampling units (PSUs) and households covered in the World Health Survey (WHS-2003) comprised the baseline sample for SAGE Wave1-India in 2007–2008. The PSUs were stratified by region and location (urban/rural) and, within each stratum, enumeration areas were selected. The enumeration areas in rural and urban areas were villages and Census Enumeration Blocks (CEBs), respectively. Within each selected areas lists of households with at least one member aged 45 and above was prepared. From the list of households, 28 households in each village and 33 household in each CEBs were randomly selected. The details are also given on the SAGE website and published elsewhere (27, 28). It was a cross-sectional household survey, which collected information for adults aged 18 years and above by face-to-face interviews. A cross-sectional study is an observational study. The observations were recorded at a singular point of time without influencing the respondents. Household and individual weights were post-stratified to weight up to population distributions by age and sex. A physical examination was used to collect height, weight, waist circumference, and blood pressure (28). Tobacco-related information was collected from the respondent who reported consuming tobacco. The total sample size was 4,038 respondents who were the current consumer of any form of tobacco.

### Tobacco Use Classification

The tobacco users reported consuming only smokeless tobacco was categorized as smokeless tobacco users. The respondents who reported only smoked tobacco were categorized as smokers. The respondents who reported using both the product were categorized as user of “both” product. The smokeless tobacco user and smokers were classified by duration of tobacco use, i.e., using less than for 10 years and using it for 10 and more years. Smoking was also categorized by daily uses. Smoking less than 10 U per day were classified as mild/moderate smokers, smoking 10 or more units per day were categorized as severe smokers. Similarly, Smokeless tobacco users consuming less than 10 g per day were classified as a mild/moderate use. Smokeless tobacco users consuming 10 or more grams per day categorized as severe use.

### Morbidity Indicators

Systolic and diastolic blood pressure levels were recorded three times for each respondent. The average of these three systolic and diastolic blood pressures was taken as the final systolic and diastolic blood pressure level of the respondent. They were then categorized as hypertensive if systolic blood pressure was greater than 140 mmHg or diastolic blood pressure greater than 90 mmHg. Height and weight of the respondents taken during the survey were used to calculate body mass index (BMI) as per the following formula:

$$\text{BMI}=\frac{\text{Weight}\left(\text{kg}\right)}{\text{Height}{\left(M\right)}^{2}}.$$

The classification of BMI followed the WHO standard, i.e., ranging from BMI less than 18.5 which was categorized as underweight to BMI greater than 25.0 which was overweight. Self-reported morbidities such as asthma, chronic lung disease (CLD), diabetes mellitus, hypertension, and stroke were also analyzed.

### Statistical Analysis

The bivariate analysis was used to see the distribution of tobacco use. The prevalence of chronic morbidities was estimated for smokers and smokeless tobacco users. Missing values were excluded from the analysis. The estimates were weighted using the 2001 Indian Census population for the age–year-specific distribution of the population. The risks (odds ratios) of each chronic condition were estimated for smokeless tobacco users, taking smoking as the reference category. The odds ratio for hypertension with no education and hypertensive with poor household (belonging to lowest wealth quite) was also estimated. The odds ratio was adjusted for age, gender, place of residence, religion, caste, education, wealth quintile, and states. The logistic regression was used to estimate the odds ratio. The outcome variables were binary and logistic regression is appropriate for binary outcomes. Logistic regression converts the binary outcome into probability and generates odds ratios for each response variables. Data analyses were performed using STATA version 12.0 software.

## Results

Table 1 shows the socioeconomic distribution of the sample. A high percentage of smokeless tobacco users and smokers belong to higher age groups (aged above 50 years). More than 80% of the sampled tobacco users (both smoker and smokeless) belong to rural areas. Almost 80% of smokers were males, while around half of smokeless tobacco users were males. Education was also similar in both of the tobacco users. Except gender, all the other socioeconomic characteristics are similar for both of the tobacco user in our sampled population.

**Table 1:** Socioeconomic characteristics of the sampled population.

Socioeconomic characteristics	Smoking		Smokeless		Both (smoking and smokeless)	
	*N*	%	*N*	%	*N*	%
**Age groups**						
18–29	38	2.8	143	6.1	13	4.0
30–49	281	20.9	573	24.6	70	21.3
50–59	472	35.1	708	30.4	107	32.6
60–69	362	26.9	545	23.4	94	28.7
70 and above	192	14.3	358	15.4	44	13.4
**Place of residents**						
Urban	235	17.5	437	18.8	51	15.5
Rural	1,110	82.5	1,890	81.2	277	84.5
**Gender**						
Male	1,130	84.0	1,145	49.2	292	89.0
Female	215	16.0	1,182	50.8	36	11.0
**Religion**						
Muslim	192	14.3	327	14.1	41	12.5
Hindu	1,129	83.9	1,914	82.3	278	84.8
Others	24	1.8	86	3.7	9	2.7
**Caste/tribe**						
Scheduled caste/tribe	379	28.4	701	30.6	98	29.9
Others	957	71.6	1,592	69.4	230	70.1
**Education**						
No education/less than primary	807	60.0	1,464	62.9	182	55.5
Primary	227	16.9	351	15.1	61	18.6
Secondary	135	10.0	251	10.8	43	13.1
Higher secondary and above	176	13.1	261	11.2	42	12.8
**Wealth quintile**						
Lowest	284	21.1	542	23.6	75	22.9
Second	341	25.4	520	22.6	64	19.6
Third	253	18.8	456	19.8	86	26.3
Fourth	228	17.0	460	20.0	60	18.3
Highest	238	17.7	323	14.0	42	12.8
**States**						
Assam	65	4.8	470	20.2	40	12.2
Karnataka	154	11.4	278	11.9	29	8.8
Maharashtra	79	5.9	407	17.5	33	10.1
Rajasthan	415	30.9	219	9.4	60	18.3
Uttar Pradesh	295	21.9	512	22.0	109	33.2
West Bengal	337	25.1	441	19.0	57	17.4

**Figure 1:**
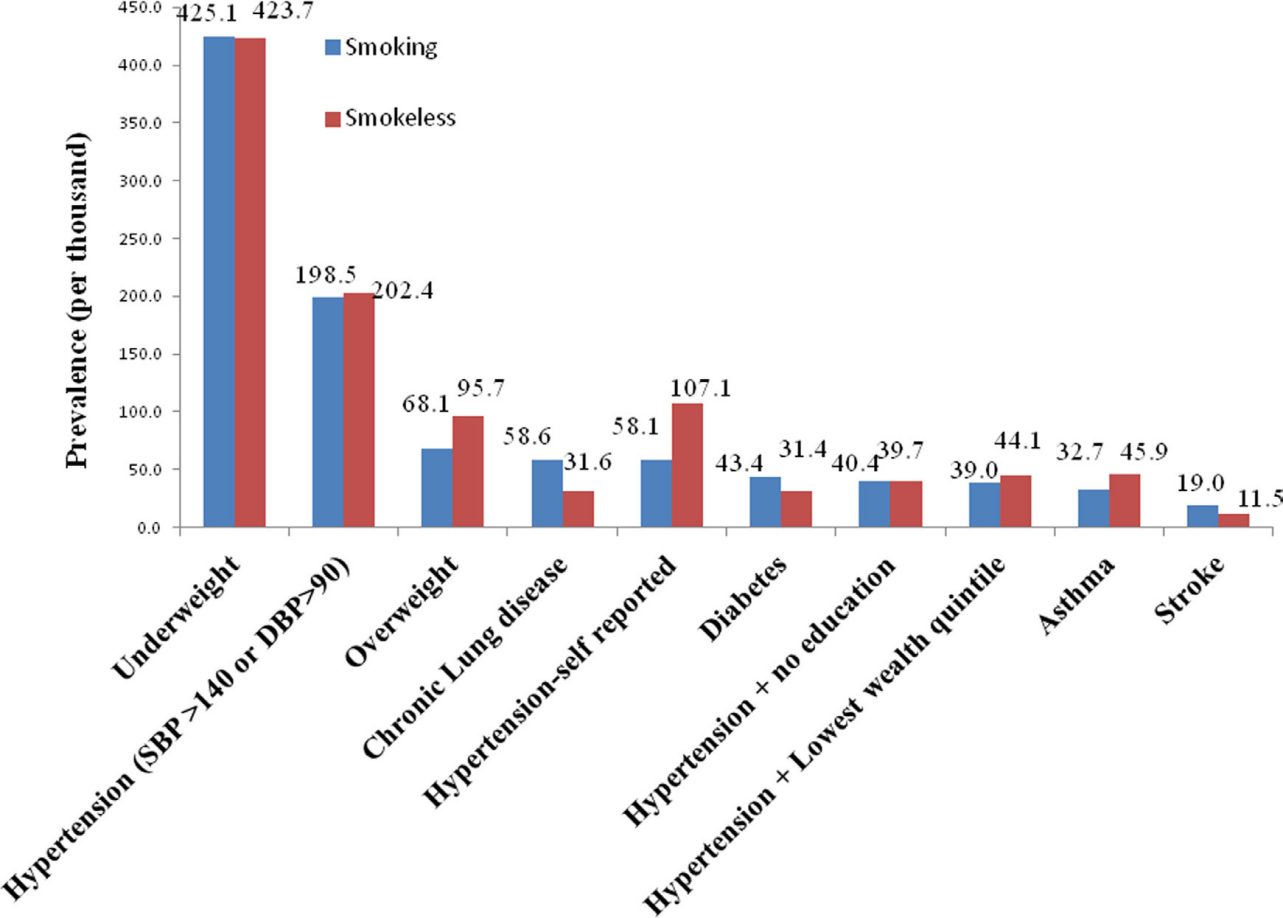
Prevalence of morbidities (per thousand) among smokers and smokeless tobacco users.

The prevalence of hypertension (self-reported) was higher for smokeless tobacco users but the prevalence of hypertension based on blood pressure levels was almost same for smokers and smokeless tobacco users (Figure 1) the prevalence of diabetes, stroke, and CLD was higher for smokers. The prevalence of asthma and underweight was higher for smokeless tobacco.

The odds ratios of chronic diseases were estimated taking smoking as the reference category (Table 2). Adjusted odds ratios for diabetes, asthma, hypertension (self-reported and measured) for smokeless tobacco user were compared to smoking and the differences were not statistically significant. The odds ratio of hypertension for low education and for people belonging to lowest wealth quintiles household was also not significant, except for CLDs. The risk of CLD was less among smokeless tobacco users compared to smokers. A similar finding has also been found for duration and amount of smokeless tobacco user (Tables 3 and 4). The odds ratio of hypertension was not significant for smokeless tobacco users consuming less than 10 g per day as well as consuming 10 g or more when compared to smoking (less than 10 U per day). There was also no significant difference found for the odds ratio of hypertension when compared to the duration of smokeless tobacco use with smoking (Table 4).

**Table 2:** Odds ratio (95% CI) of chronic diseases comparing smokeless tobacco with smoking.

	Smokeless		Both (smoking and smokeless)		Pseudo *R*-square
	OR (95% CI)	*p*-Value	OR (95% CI)	*p*-Value	
Stroke	0.73 (0.43–1.24)	0.372	0.73 (0.28–1.93)	0.575	0.056
Diabetes	1.17 (0.82–1.67)	0.776	1.00 (0.55–1.83)	0.980	0.068
Chronic lung diseases (CLDs)	0.64 (0.45–0.91)	0.040	0.72 (0.41–1.27)	0.183	0.053
Asthma	1.21 (0.89–1.64)	0.131	0.74 (0.42–1.30)	0.304	0.049
CLDs and Asthma	1.04 (0.77–1.31)	0.950	0.80 (0.51–1.23)	0.310	0.052
Hypertension	1.24 (0.98–1.58)	0.359	0.93 (0.60–1.44)	0.656	0.080
Systolic BP > 140	1.17 (0.95–1.43)	0.259	0.84 (0.58–1.21)	0.438	0.049
diastolic BP > 90	1.12 (0.93–1.34)	0.398	1.15 (0.86–1.54)	0.165	0.013
Hypertension (systolic BP > 140 or diastolic BP > 90)	1.13 (0.96–1.34)	0.352	1.09 (0.83–1.45)	0.299	0.019
Underweight	0.79 (0.68–0.92)	0.035	1.01 (0.78–1.31)	0.624	0.602
Overweight	1.07 (0.81–1.40)	0.391	0.71 (0.42–1.20)	0.292	0.078
Hypertensive + no education	1.33 (0.92–1.91)	0.554	1.46 (0.85–2.52)	0.143	0.022
Hypertensive + poor	1.12 (0.80–1.56)	0.548	1.42 (0.85–2.38)	0.277	0.067

*Reference category is smoking*.

**Table 3:** Odds ratio (95% CI) of chronic diseases comparing amount of smoking and smokeless tobacco consumption.

	Smoking (10 years and above)		Smokeless (less than 10 years)		Smokeless (10 years and more)		Both (smoking and smokeless)		Pseudo *R*-square
	OR (95% CI)	*p*-Value	OR (95% CI)	*p*-Value	OR (95% CI)	*p*-Value	OR (95% CI)	*p*-Value	
Chronic lung diseases (CLDs)	1.08 (0.67–1.73)	0.763	0.75 (0.47–1.17)	0.206	0.55 (0.25–1.23)	0.144	0.69 (0.37–1.32)	0.266	0.061
Asthma	0.77 (0.48–1.22)	0.268	1.22 (0.83–0.79)	0.319	1.03 (0.55–1.93)	0.920	0.66 (0.35–1.21)	0.178	0.046
CLDs and asthma	0.88 (0.61–1.27)	0.485	0.98 (0.71–1.36)	0.909	0.75 (0.43–1.29)	0.292	0.75 (0.46–1.20)	0.225	0.054
Hypertensive (Systolic BP > 140 or diastolic BP > 90)	0.92 (0.60–1.40)	0.349	0.72 (0.46–1.14)	0.794	1.12 (0.74–1.70)	0.641	1.09 (0.80–1.49)	0.573	0.015
Hypertensive + no education	0.88 (0.24–2.28)	0.885	1.01 (0.37–2.79)	0.918	1.22 (0.48–3.09)	0.451	1.16 (0.46–1.53)	0.548	0.020
Hypertensive + poor	1.24 (0.48–3.22)	0.379	1.64 (0.62–4.31)	0.241	1.32 (0.52–3.34)	0.39	1.07 (0.45–2.55)	0.688	0.066

*Reference category is smoking (less than 10 years)*.

**Table 4:** Odds ratio (95% CI) of chronic diseases comparing duration of smoking and smokeless tobacco consumption.

	Smoking (10 U per day and more)		Smokeless (less than 10 g per day)		Smokeless (10 g per day and more)		Both (smoking and smokeless)		Pseudo R-square
	OR (95% CI)	*p*-Value	OR (95% CI)	*p*-Value	OR (95% CI)	*p*-Value	OR (95% CI)	*p*-Value	
CLDs	1.31 (0.46–3.75)	0.619	1.21 (0.40–3.71)	0.734	0.80 (0.28–2.29)	0.675	0.86 (0.28–2.30)	0.675	0.082
Asthma	1.20 (0.42–3.41)	0.738	2.00 (0.68–5.89)	0.208	1.53 (0.54–4.32)	0.423	0.90 (0.29–2.84)	0.863	0.067
CLDs and Asthma	1.48 (0.63–3.50)	0.372	1.79 (0.73–4.37)	0.203	1.38 (0.5.9–3.25)	0.459	1.17 (0.46–2.95)	0.741	0.054
Hypertensive (systolic BP > 140 or diastolic BP > 90)	0.85 (0.66–1.10)	0.204	1.04 (0.85–1.28)	0.828	0.99 (0.71–1.36)	0.952	0.88 (0.54–1.42)	0.596	0.0126
Hypertensive + no education	0.87 (0.50–1.52)	0.459	1.29 (0.82–2.51)	0.465	0.94 (0.45–1.98)	0.865	1.50 (0.52–4.31)	0.45	0.0199
Hypertensive + poor	1.12 (0.66–1.87)	0.613	1.17 (0.76–1.80)	0.339	1.22 (0.65–2.31)	0.569	1.50 (0.54–4.21)	0.44	0.0665

*Reference category is smoking (less than 10 U per day)*.

## Discussion

Globally, smoking is one of the leading causes of premature mortality (29, 30). The reports also suggest that the impact of smoking have been subdued in low-middle-income countries compared to high-income countries (12, 13, 31). There had been a little debate over the impact of smokeless tobacco use on chronic diseases, due to variability in the type of smokeless tobacco products available globally as well as within countries. Smokeless tobacco is the most common form of tobacco use in India (8, 32, 33). Smokeless tobacco use is very common among women tobacco users, which is similar to other studies (9, 34, 35). Many studies have found statistical significant differences for smokers and smokeless tobacco users on non-communicable disease incidence compared to non-tobacco users (19, 35, 36). We found only the odds ratio of CLD to be significantly lower in smokeless tobacco users compared to smoked tobacco users. Smoking is related to an increased risk of type 2 diabetes compared to non-smokers (36). Studies in Sweden have observed higher risk of stroke among smokeless tobacco user compared with non-tobacco user (22). The mortality due to CVDs has also reported higher among smokeless tobacco users compared to non-tobacco users (21). Studies in India have also exhibited higher chances of chronic illness among smokeless tobacco compared with non-tobacco users (25, 26). We did not found studies comparing smokeless with smoking in the context of chronic illness. Our study adds to the previous finding, that there are no less risk of chronic diseases for smokeless tobacco users compared to smoking.

The chemical composition of both form of tobacco is very similar (6). However, chemical properties due to burning of the tobacco may have contributed to higher risk of lung related diseases (6, 7). Chewing tobacco can be related to more exposure to the chemicals causing cancers related with mouth and throat (7). Studies have also found that use of less harmful smokeless tobacco is related to more risk of smoked tobacco (37). Therefore, smokeless tobacco cannot be used as cessation tool for smoking (37).

We have also not found significant influence on non-communicable diseases by duration and amount of tobacco consumption, comparing smoking and smokeless tobacco users. Our study suggests that the use of smokeless tobacco may be as harmful as smoking in the context of chronic diseases. As we know that the smokeless tobacco is the most common form of tobacco consumed in India (8, 34, 35). The policies preventing tobacco epidemic must focus on containing the use of smokeless tobacco along with smoking.

There are several limitations of the study. The analysis is based on the cross-sectional survey; based on secondary data, the causal relationships cannot be established. Data on various types of smokeless tobacco use were not available; the risks may also differ by types of smokeless tobacco. Some of the chronic conditions and tobacco use were self-reported, which may influence the result.

## Conclusion

The study has shown that there is no difference for the risk of chronic conditions between smoked and smokeless tobacco users. The risk of hypertension was also similar in both form of tobacco when comparing amount and duration of smoking with smokeless tobacco consumption. As the smokeless tobacco is the most common form of tobacco used in India, the policy must address in decreasing as well as preventing the use of any form of tobacco in India.

## Ethics Statement

The study is based on secondary data analysis. No data were collected for this study. The data are available for free on the WHO (World Health Organization) website. The WHO-SAGE 2007-08 study is approved by WHO and their partner organization in various countries (www.who.int/healthinfo/systems/sage). There is no need for ethical clearance.

## Author Contributions

AA has contributed toward conceptualization of the study, data analysis and writing the manuscript. MS has contributed toward data analysis, writing, and revising the manuscript.

## Conflict of Interest Statement

The authors have declared that there are no competing interests exist in the preparation of this article.

## Abbreviations

CVD, cardiovascular diseases, WHO, World Health Organization, BMI, body mass index, SAGE, Study on Global Ageing and Adult Health, PSU, primary sampling units.
